# The construction of mine water recycling performance evaluation index system under the Internet of Things environment

**DOI:** 10.1038/s41598-023-37224-8

**Published:** 2023-06-26

**Authors:** Mingzhe Lei, Yang Li, Ning Zhou, Yue Zhao

**Affiliations:** 1 CHN Shendong Coal Group Co., LTD., Shenmu, 719300 China; 2 Summit Technologies Co., LTD., Xi’an, 710000 China

**Keywords:** Biochemistry, Biological techniques, Ecology, Biogeochemistry, Environmental sciences, Environmental social sciences, Hydrology, Limnology

## Abstract

The utilization rate of water resources of mines in China is still relatively low. The evaluation of mine water recycling has practical guiding significance for the planning, positioning, development, and construction of groundwater in today’s society. This article constructs an evaluation system for mine water recycling based on the key performance index (KPI) via the Internet of Things and big data platforms. This system evaluates the recycling status of mine water. First, the micro-seismic monitoring system and the hydrological dynamic detection system are deployed in work. The installation and debugging methods are compared to meet the monitoring requirements. Second, the filtered clear water is used for equipment cooling and firefighting dust removal at the mining face through the constant pressure supply pump. The excess clear water is discharged to the surface. Finally, 16 indicators are screened from four dimensions to construct a key KPI mine water evaluation system for evaluation and optimization. The results demonstrate that the first mine water monitoring system runs well and is fully functional, achieving the expected goal. The utilization rate evaluation score has increased yearly, from 3.05 points in 2016 to 3.39 points in 2020. However, the per capita utilization rate score still needs improvement. It is essential to improve the rationality of development and utilization.

## Introduction

The treatment of mine water in China commenced in the mid-1980s and has since witnessed remarkable progress and technological advancements. Currently, the majority of registered mining enterprises nationwide, with the exception of a few water-free mines in the northwest region, have established and operated mine water treatment systems. Based on incomplete statistics and estimates, the total treatment capacity for various types of mine water in the country exceeds 5 billion cubic meters per year. With the exception of a few mines employing pre-drainage or underground diversion measures, enabling direct discharge of clean mine water, most mines necessitate water quality treatment^1^. Achieving comprehensive recycling and utilization of mine water has become an essential aspect of cost reduction and efficiency enhancement in mine operations. As a result, ensuring efficient and cost-effective comprehensive utilization of mine water poses a significant challenge for mine management and technical personnel.

Since the introduction of the Internet of Things (IoT) concept, substantial theoretical advancements have been made, and significant economic and social benefits have been realized through its application in various domains, including transportation, logistics, electricity, healthcare, agriculture, and urban management. Drawing inspiration from the extensive implementation of the IoT, the concept of smart water management, referred to as “Smart Water Conservancy”, has emerged to address the needs of mine water resource supply, demand, and distribution^2^. However, mine water differs from logistics, and the dual depiction of water flow concerning “field” and “quality” traditionally renders water networking more intricate and challenging in comparison to the IoT. Smart water management, based on IoT principles, enables real-time perception, process tracking, and dynamic simulation of the complete mine water circulation process. By integrating information fusion and data mining of both market water networks and physical water networks, it optimizes the allocation and intelligent supervision of mine water resources, thereby enhancing the efficiency of mine water utilization^3^.

This article aims to propose a comprehensive and universally applicable performance evaluation index system for mine water circulation, guided by the concepts of a healthy city and mine water circulation. By utilizing detailed, quantifiable, and widely applicable performance evaluation indicators, the article adheres to the principle of performance assessment and seeks to establish a generalized evaluation system. Firstly, this article elucidates the operation of existing microseismic monitoring systems and hydrological dynamic detection systems. Secondly, the filtration process is accomplished by leveraging the water storage capacity of goafs and the height difference of tunnels, facilitating the natural flow of wastewater within goafs. The filtered clean water is then utilized for equipment cooling and fire dust removal in the mining working face using a constant pressure water supply pump, while any excess clean water is discharged to the surface. Finally, 16 key performance indicators (KPIs) are selected from four dimensions: ecological level, water resource abundance, water resource quality, and water resource utilization. These indicators form the evaluation system for mine water circulation, serving the purpose of assessment and optimization.

## Literature review

Extensive research has been conducted by numerous scholars on issues related to urban water circulation. For instance, Hou elaborated on the developmental process of urban hydrology, with a particular focus on the impacts of urbanization on various aspects of the water cycle, including precipitation, runoff, infiltration, and evapotranspiration^4^. Manikandan introduced the concept of social water circulation and examined the sustainability of urban water resources, establishing an evaluation system for the environmental sustainability of urban water systems^5^. Pinotti investigated the transformation process of urban water circulation and explored the necessary conditions for a well-functioning water system cycle, proposing a rational application sequence for urban water resources^6^. Scanlon discussed the connection between modern socioeconomic factors and sustainable water resource utilization, suggesting a range of measures to promote urban water circulation within a circular economy framework^7^.

Research and application of IoT technologies in the realm of smart water management primarily focus on real-time perception, water information interconnection, process tracking, and intelligent processing. Ighalo utilized IoT data acquisition and environmental sensing technologies to study and enhance the timeliness and accuracy of data acquisition from various water monitoring devices^8^. Friha leveraged the achievements of IoT in intelligent identification, tracking positioning, and monitoring management to achieve refined management of basin water resource allocation processes, water environment changes, hydrological element evolution, and other water-related factors^9^. Yasin utilized the IoT's capabilities to acquire and process data from diverse business systems in a dynamic, open, and uncontrollable environment, enabling water information sharing in a watershed context^10^. Sinha capitalized on the research findings of IoT in industrial intelligent control, domain knowledge processing, and big data analysis to improve the efficiency and accuracy of water-related business processes, progressively realizing smart water management^11^.

Okudan, through an analysis of urban water circulation processes and drawing from the principles of a key performance indicator system, developed an evaluation index system for the health assessment of urban water circulation systems. This evaluation index system facilitated the calculation of indicator weightings through modeling, enabling the quantitative analysis of the health status of water circulation in Tianjin. The analysis provided insights into the health conditions of Tianjin's water circulation across different levels, aspects, and years^12^. D'Inverno, considering the natural and social characteristics of urban water circulation, focused on the processes of water supply, water use, wastewater drainage, and reuse. By examining their interrelationships and coordination, a health evaluation system for urban water circulation was established^13^. Zhang, taking into account the characteristics of water resources in Xi’an, employed a subjective–objective weighting method and selected 16 indicators from four dimensions. Using a rating description method with five levels of evaluation, a key performance indicator-based evaluation system for the health of water circulation in Xi'an was constructed, providing theoretical support for water resource management^14^.

However, previous studies have focused mostly on the specific water resource cycle, cycle sustainability, and water cycle law. Scholars have examined some technologies of coal mines in many regions, combined with the mine’s hydrogeological conditions and actual production conditions. Innovatively, the filtered sewage from the mining area is supplied directly to the underground for firefighting and cooling by adding constant pressure water supply equipment. This solution realizes the underground recycling of mine water, effectively reduces mine production costs, and increases mine efficiency. In addition, 16 indicators are selected from four dimensions using subjective and objective assignments. Besides, a five-level water cycle health evaluation based on key performance indicators is built using the hierarchical description method. This system provides theoretical support for water resources management.

## Model design

**Figure 1:**
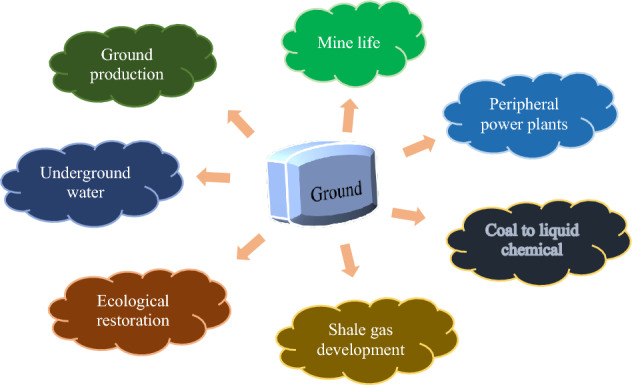
Applications of the mine water industry.

In the coal mining process, groundwater is in contact with coal seams and rock formations. During the coal mining process, a series of physical, chemical, and biochemical reactions occur due to the contact between groundwater and coal and rock seams, with the influence of human activities. Consequently, the water quality has significant coal industry characteristics. Mine water with poor sensory properties has a much higher suspended matter content than surface water. It contains suspended matter with small particle size, light-specific gravity, slow settling speed, poor coagulation, and organic pollutants, such as waste machine oil and emulsified oil^15^. Mine water contains a much higher content of total ions than normal surface water. Among them, sulfate ions account for a large proportion. Mine water often has a deficient pH value. It is often accompanied by a large number of ferrous ions, increasing the treatment difficulty^16^. Figure 1 presents the industrial application of mine water.

**Figure 2:**
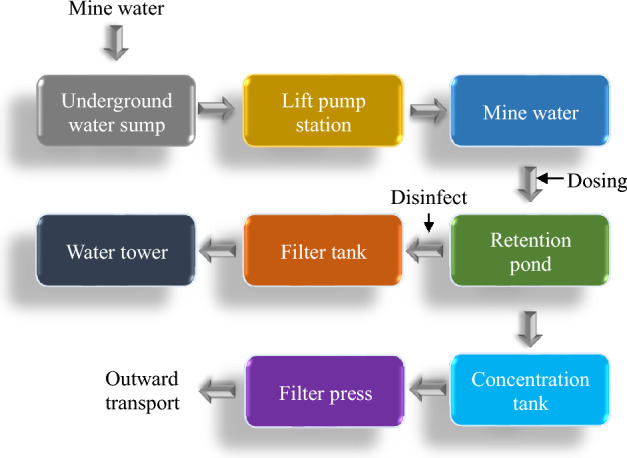
Basic process flow of traditional mine water treatment.

The pending mine water can be divided into general suspended matter mine water, high salinity mine water, acid mine water, and clean mine water. The categories are the basis for selecting a proper mine water quality treatment process. In addition, mine water is affected by human activities as it flows through mining workings, roadways, and extraction areas. Meanwhile, rock dust, coal dust, and other organic matter enter the water body, making the water quality complex^17^. Therefore, the resource utilization of mine water should adopt different treatment processes according to different types of mine water. Figure 2 displays the basic process flow of traditional mine water treatment.

The key is the development and application of an automatic dosing system and the selection of appropriate coagulants. These operations can save chemicals, simplify processes, improve effluent quality, and realize the recycling of mine water resources^18^.

**Figure 3:**
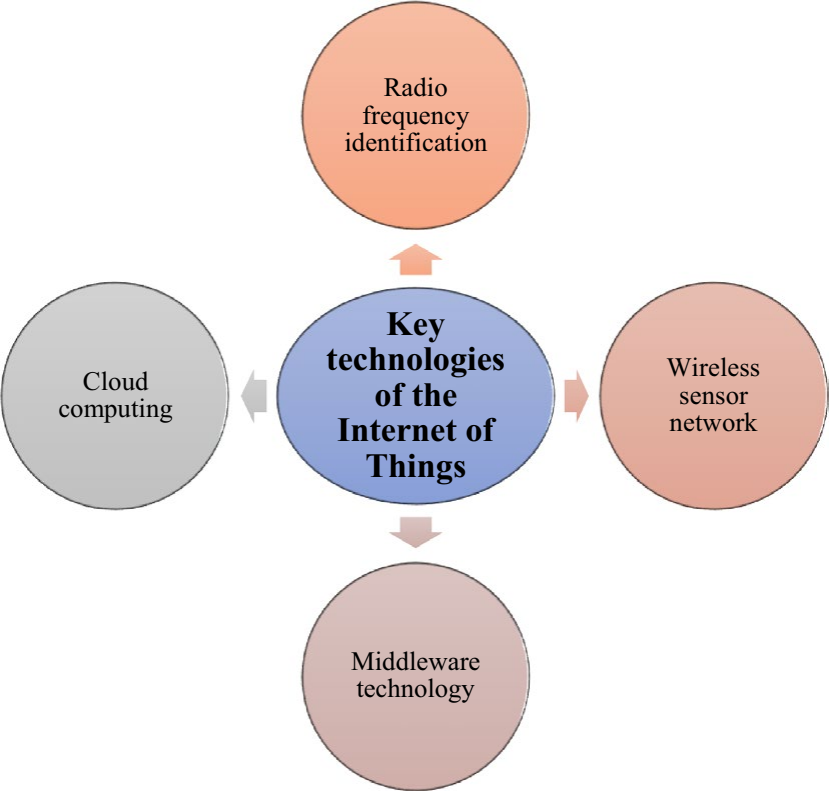
Composition of the key IoT technologies.

The development of IoT, cloud computing, and big data technology has injected new vitality into the informatization of water conservancy. IoT is characterized by sensing, interconnection, and intelligence. Its wide application has dramatically improved the standardization, efficiency, and ease of use of information services in various industries and pushed water conservancy information into a new stage. At this stage, problems such as water resource shortage and environmental pollution become even more prominent. Floods and droughts caused by extreme weather significantly impact social and people’s livelihoods, and the traditional water conservancy industry faces severe challenges^8^. The development and application of IoT-related technologies provide effective measures for intelligently solving water conservancy problems. Figure 3 illustrates the composition of its key technologies.

The transformation of IoT technology from concept to the practical application requires integrating several key IoT technologies. Common techniques include radio frequency identification technology, wireless sensor technology, middleware technology, and cloud computing technology. The integration of these technologies enables the application of IoT technology in many industries and provides the necessary conditions for the intelligence of the sector^19^.

**Figure 4:**
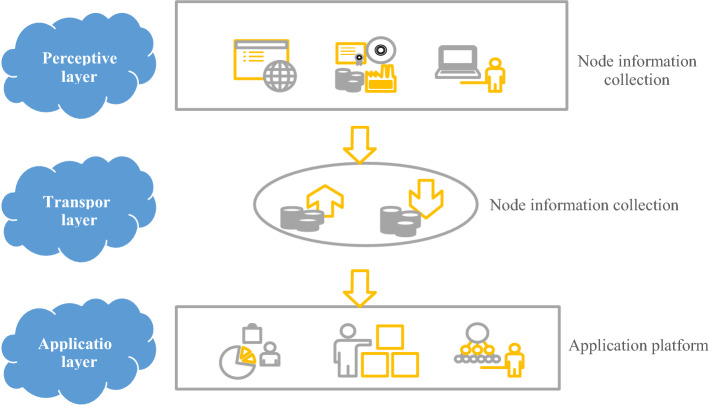
Constitute of the mine water resource smart network.

This article uses IoT technology to build a smart mine water resource network. It applies water resources-related sensors (for metering facilities, water quality, water level, flow monitoring, etc.). This network is conducive to mine water resources development, utilization, and protection. Then, the sensors of each node are connected to the existing wireless network and the Internet. The intelligent mine water resources network is mainly composed of three levels of systems, as shown in Fig. 4.

**Figure 5:**
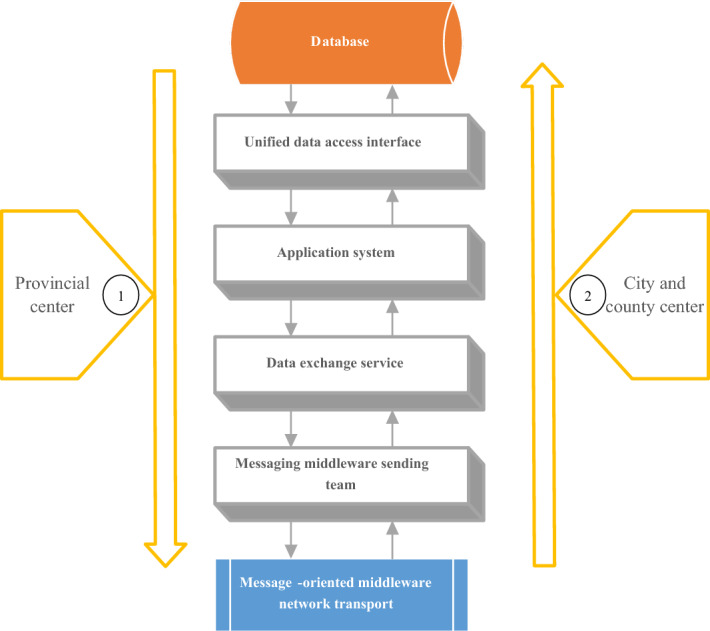
Data transmission process of the mine water resource smart network.

The intelligent mine water resource network consists of three layers: the perception layer (node information collection), the transmission layer (information transmission), and the application layer (the application platform). The perception layer mainly collects information such as water volume, temperature, level, and quality to form each node of the intelligent network of mine water resources. The transmission layer transmits the information collected by each node to the application platform. As a platform for data storage, processing, analysis, and application, the application layer provides application software for mine water resources management^20^. The intelligent mine water resource network depends on the synergy of three levels of perception, transmission, and application. It realizes intelligent monitoring, measurement, scheduling, and management functions of the mine water resources environment. Figure 5 reveals the data transmission process.

The intelligent mine water environment monitoring system acquires the primary quality and quantity data of key water function areas. Then, it calculates according to the water resource consumption of each water intake point and real-time data sent back from the remote monitoring network. The water quality and quantity information of mine water resources and the water supply and demand status can be grasped in real time according to the analysis results. In this way, the intelligent scheduling of water resources is realized to provide services for developing, utilizing, allocating, and protecting water resources^21^.

### Analytic hierarchy process

An index evaluation system is established to stratify complex problems. The importance of each indicator is judged by constructing a judgment matrix of indicators at the same level. Here, the 1–9 scale is used to obtain the judgment matrix, as shown in Eq. (1).

1 $$S={({u}_{ij})}_{n\times n}$$

The largest eigenvalue ${\lambda }_{max}$ and the eigenvector $W$ of the judgment matrix of each level are solved. The weight of the level index is obtained by normalizing the feature vector:

2 $$W=[{\omega }_{1}^{\prime},{\omega }_{2}^{\prime},\dots ,{\omega }_{n}^{^{\prime}}]$$

3 $$CI=\frac{{\lambda }_{max}-n}{n-1}$$

4 $$CR=\frac{CI}{RI}$$

where ${u}_{ij}$ signifies the scale of the indexes $i$ and $j$; ${\omega }_{i}^{\prime}$ refers to the index weight of $i$; $CI$ denotes the consistency index; $CR$ represents the consistency ratio; $RI$ indicates the random consistency index; $n$ stands for the number of paired comparison factors. If *CR* < 0.1, the consistency check is passed; otherwise, the judgment matrix is rebuilt. Then, the weight of each indicator to the highest level is calculated using the weight of each indicator in the same layer and the weight of the previous layer.

### Entropy method

The entropy method can effectively eliminate the influence of subjective factors. The greater the system information entropy, the more balanced the system structure, the smaller the difference, and the smaller the weight^22^. On the contrary, the more unbalanced the system structure, the greater the difference and the greater the weight. The specific calculation steps are as follows.

The weight of the *j*th indicator in the *k*th year is calculated according to Eq. (5).

5 $${X}_{kj}={x}_{kj}/\sum_{k=1}^{m}{x}_{kj}$$

The index information entropy can be written as Eq. (6).

6 $${E}_{j}=-\frac{1}{Inm}\sum_{k=1}^{m}{x}_{kj}{X}_{kj}$$

Equation (7) describes the information redundancy.

7 $${D}_{J}=1-E$$

Equation (8) indicates the index weight.

8 $${\omega }_{j}^{\prime \prime}={D}_{j}/\sum_{j=1}^{n}{D}_{j}$$

In Eq. (8), $m$ represents the year; $n$ refers to the index sample number; ${x}_{kj}$ denotes the value of the *j*th index in the *k*th year. The minimum relative information entropy principle and the Lagrange multiplier method are used to optimally determine the combination weights of each index. The combination weight of the *i*th indicator is calculated via Eq. (9).

9 $${\omega }_{i}=\frac{{\left({\omega }_{i}^{{\prime}}\times {\omega }_{i}^{\prime \prime}\right)}^{0.5}}{{\sum }_{i=1}^{n}{\left({\omega }_{i}^{{\prime}}\times {\omega }_{i}^{\prime \prime}\right)}^{0.5}},\quad (i=\mathrm{1,2},\dots ,n)$$

In Eq. (9), ${\omega }_{i}^{{\prime}}$ represents the weight of the *i*th index obtained by the analytic hierarchy process (AHP); ${\omega }_{i}^{\prime \prime}$ denotes the weight of the *i*th index obtained by the entropy method; ${\omega }_{i}$ refers to the weight of the *i*th index combination.

**Figure 6:**
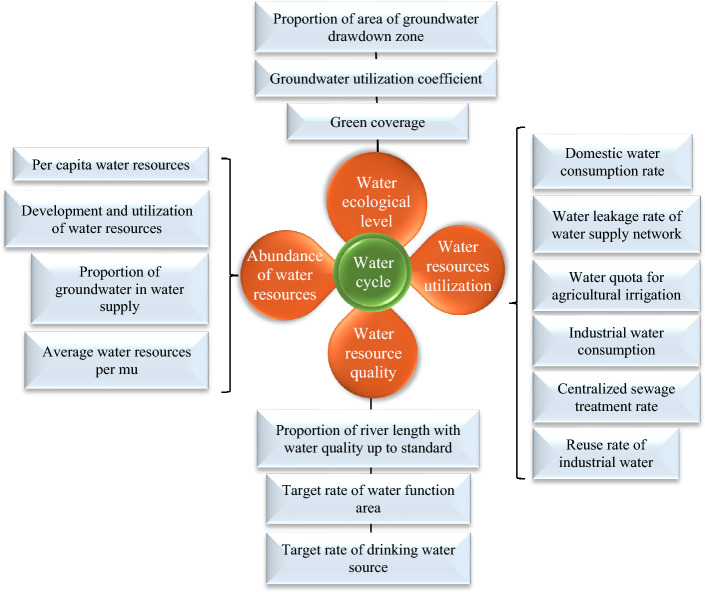
System monitoring scheme.

The mine water monitoring system consists of the mine water monitoring IoT system, mine water monitoring, and early warning software system, and mine water monitoring and early warning big data platform^23^. The mine water monitoring IoT system uses sensors of pressure, temperature, water level, and other parameters to watch the prominent monitoring locations of the mine according to the set plan. The monitoring signals are transmitted to the central ground station through the transmission substation. The central station deploys the mine water monitoring and early warning software system to process the signs of each measurement point. It provides query, printing, and early warning functions. Besides, the software system is connected to the big data platform for mine water monitoring and early warning. It implements comprehensive scheduling, information integration, data visualization, model prediction, and intelligent warning. Besides, it realizes the whole process of automatic acquisition, processing and analysis^24^. Figure 6 illustrates the system monitoring scheme.

**Figure 7:**
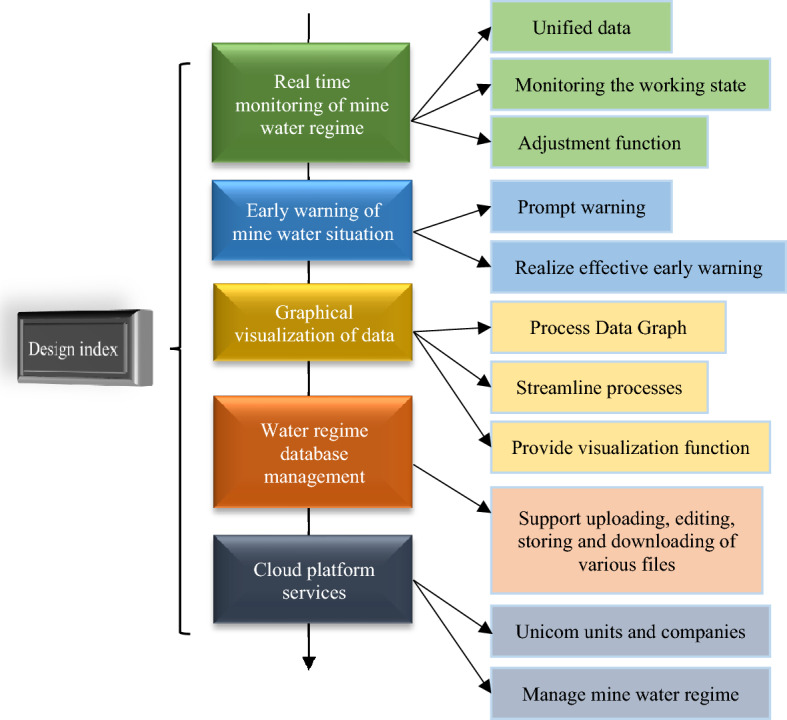
Platform design metrics.

The first step involves establishing a scientifically sound monitoring network and identifying the hydrological elements that require monitoring within the mine, thereby obtaining essential field measurement data. A comprehensive understanding of the water circulation pathways and the various equilibrium relationships within the mine can be achieved by monitoring various hydrological processes, such as groundwater recharge, drainage, agricultural water consumption, and water return. Subsequently, a water circulation process and monitoring evaluation system for the mine is constructed, enabling a systematic assessment of water resource utilization efficiency, the operational status of water engineering, and the key factors that restrict the sustainable utilization and protection of water resources. This system serves as a foundation for informed decision-making in comprehensive water resource planning and design, management and operation, rational allocation, and the pursuit of sustainable development of water resources, ultimately facilitating the establishment of an efficient and water-saving society. A big data platform for mine water monitoring and early warning is developed according to the actual needs. Figure 7 shows the leading design indicators of the platform.

**Figure 8:**
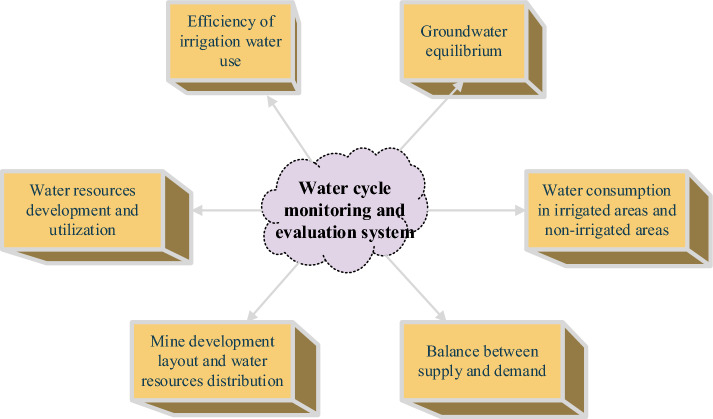
Mine water resources monitoring and evaluation system.

The monitoring data of each measuring point, the operational status of sensors, and the sensor parameters of Tianaozheng are unified. Then, the data are integrated through real-time detection to achieve effective early warning. At the same time, the monitoring data is graphically processed. Finally, the cloud service platform monitors the mine water conditions^25^. Monitoring is not an end in itself, but rather a means to achieve specific objectives. Its primary goal is to analyze, study, and evaluate the operational rationality and sustainability of the mine water circulation system through systematic monitoring. This operation, in turn, provides a scientific basis for the rational utilization of water resources and the necessary engineering support. To fulfill this purpose, this article proposes the establishment of a scientific and practical monitoring and evaluation system, as illustrated in Fig. 8.

The construction of a mine hydrological monitoring and water resources utilization evaluation system is a complex engineering endeavor. This undertaking has historically been a weak point, and it is challenging to fully implement within a short timeframe. During the implementation process, careful attention should be given to the following issues:

1. Scientific planning and rational station network layout, along with the establishment of a multi-channel investment mechanism. It is crucial to actively engage in the planning and design of the mine hydrological monitoring system, as well as the development and study of the mine water resource utilization evaluation system.
2. Strengthening industry management and standardizing monitoring practices. Mine hydrological monitoring involves multiple units working together, with a large monitoring team and varying levels of technical expertise. To ensure the reliability of monitoring results, it is necessary to implement industry management practices.
3. Actively adopting the “engineering belt monitoring” mechanism to promote the establishment of a network of mine hydrological monitoring stations. The establishment and improvement of the mine hydrological monitoring and water resource utilization evaluation system is considered a crucial indicator of a standardized and sustainable water cycle system. It should receive support in terms of construction funds and other relevant aspects.

**Figure 9:**
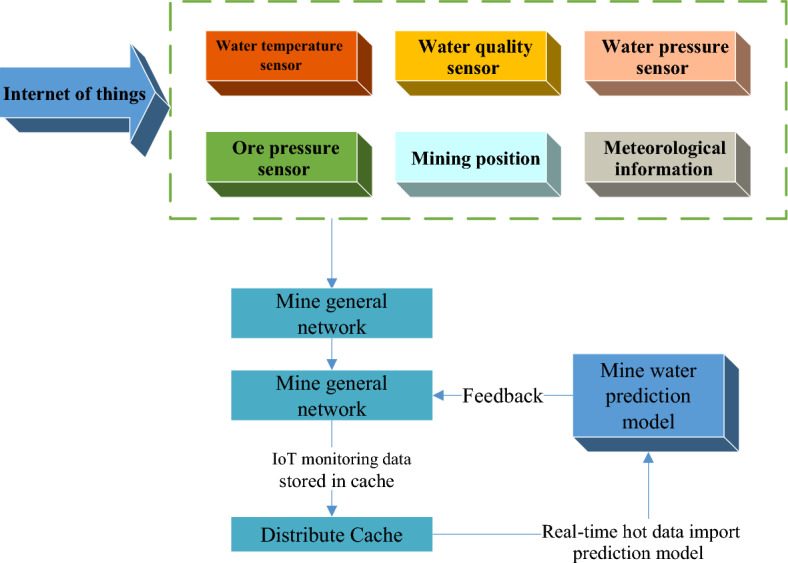
Data flow of mine water resources based on IoT.

The IoT, big data, and cloud computing are organically combined in the construction of mine water resources case base, and the real-time monitoring data of the IoT are uploaded to the big data cloud platform through the mine general network through the dispatching command center, and are temporarily stored in the form of cache. The real-time monitoring thermal data is pushed to the prediction model based on the circulating data of mine water resources, and the utilization of mine water is evaluated. Additionally, the results are fed back to the dispatching command center in real time, and the prediction model of mine water circulation is trained and updated regularly, thus forming a complete, closed and constantly improved system. The data flow trend is shown in Fig. 9:

The research significance of this paper is to clarify the actual demand of mine water recycling management and the existing water resources prevention and control norms. On the basis of extensive collection of mine water recycling management data, the mine water resource database system under the background of big data is designed and implemented in combination with technologies such as cloud computing and IoT, which provides a new idea for mine water prediction and emergency and water resources development technical training.

The water quality requirements for underground fire protection, external cooling of mining equipment, and water for fully mechanized mining face emulsion are different according to the actual mine production. Table 1 lists the specific requirements.

**Table 1:** Water use indicators.

Water use	Sewage discharge standard	Mining cooling	Fully mechanized mining face emulsion	Ventilation and dustproof
Standard status	COD value ≤ 100 mg/L	SS value (suspended solids content) does not exceed 20 mg/L	pure water	SS (suspended solids content) does not exceed 30 mg/L
	The suspended solid (SS) content does not exceed 70 mg/L	pH 7–9	The chromaticity of water quality is less than or equal to 5 degrees	Suspended matter ≤ 0.12 mm
	pH 6–9	Free Cl content 0.5–1.0 mg/L	Turbidity ≤ 1 mg/L	pH 6–9
	Ammonia nitrogen content ≤ 15 mg/L		Nothing is visible to the naked eye	
			pH 7–9	
			Conductivity ≤ 30 µs/cm	
			COD ≤ 1 mg/L	
			Hardness < 0.3 moL/L	
			Nitride ≤ 6 mg/L	

**Figure 10:**
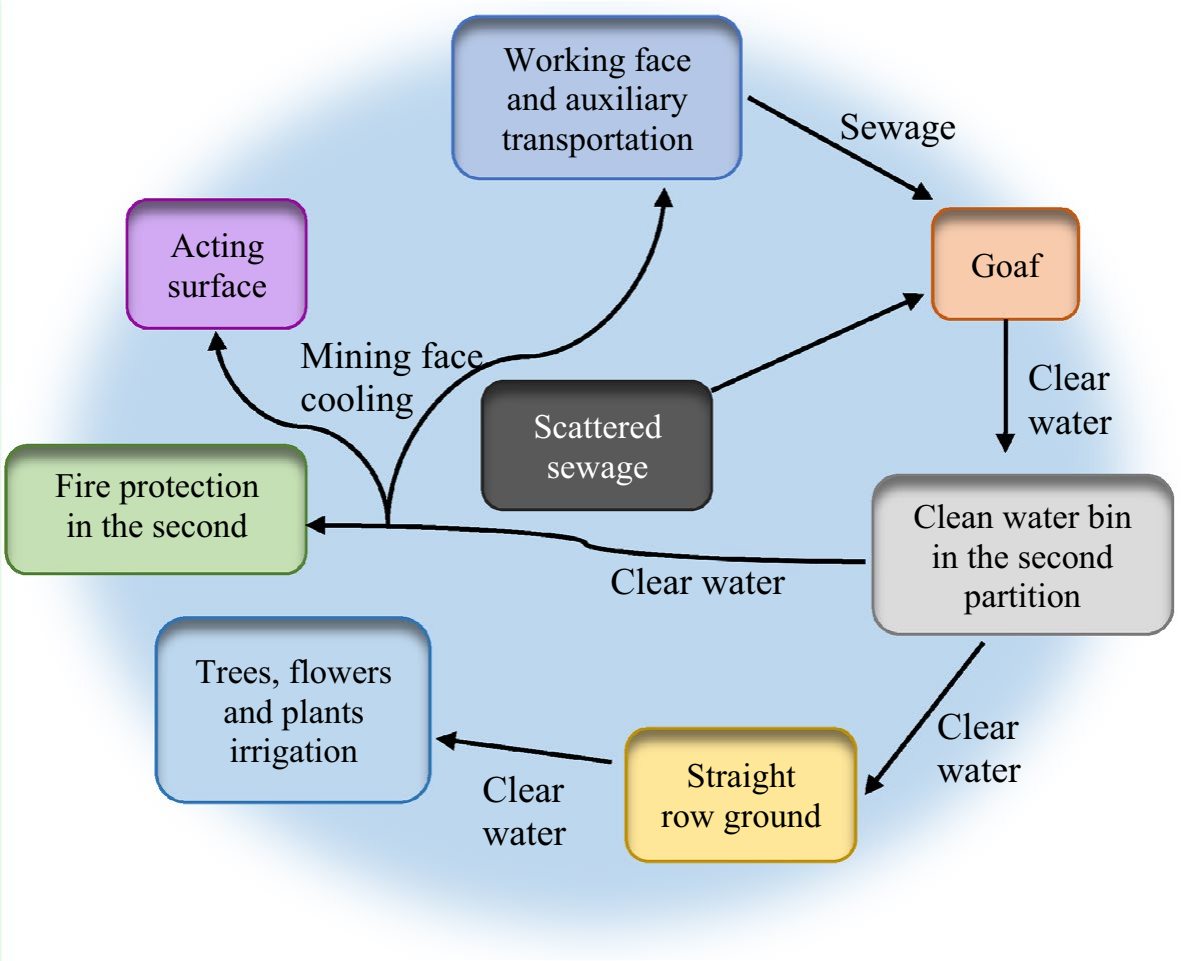
Sewage filtration and recycling process.

During the normal production of the mine, some sewage is first injected into the mining area on an experimental basis. The water quality of the water filtered through the mining area is tested. The quality of the filtered effluent meets the standard of cooling water outside the mine instead of the standard of emulsion water for header workings^26^. Figure 10 presents the process of sewage filtration and recycling.

The sewage from the lower coal seam is discharged into the second division's sewage bin through the pan zone's water bin. Then, it is injected into the joint lane closed area for filtration through the pump. In this way, the recycling of underground sewage filtration is realized. The excess part is straight on the ground for tree irrigation, bathing, etc.

**Figure 11:**
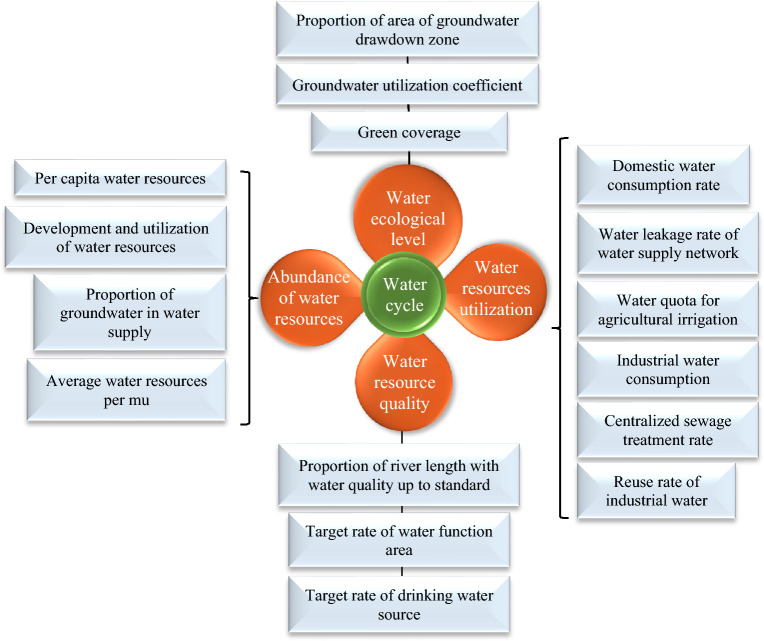
Evaluation index system.

This article obeys the principle of refined indicators, easy quantification, and promotion based on the above wastewater treatment and recycling. Besides, an evaluation system is proposed to promote mine water circulation by referring to the KPI assessment method^27^. The dimension layer is constructed based on the concepts of the “nature-society” dual water cycle and the mine water cycle. It aims at four aspects: ecological water level, water resource abundance, water resource quality, and water resource utilization. The construction of the index layer is mainly to optimize and analyze the applicability of the indicators. Specific evaluation indicators are obtained based on water quality, water quantity, water use structure, water use efficiency, and hydrological ecological environment, combined with the connotation of the water cycle^28^. This index system has certain universality in the evaluation of mine water recycling. Figure 11 indicates the specific evaluation index system.

This article combines AHP and the entropy value method to determine weights by integrating the subjective and objective variety of assignments. Firstly, the subjective weight value and objective weight value are calculated by the AHP method and the entropy value method. Then, the combined weight of the indicators is calculated by the minimum relative information entropy principle. This operation avoids the limited distribution of indicator weights caused by a single weighting, thus affecting the evaluation accuracy. Table 2 summarizes the five grades of the water recycling score based on the principles of high-quality water supply, water up to standard, clean drainage, and recycling.

**Table 2:** KPI rating scale.

Mine water recycling KPI classification					
Type	Parameter				
Evaluation standard	Very talented	Excellent	Generally	Serious	Very serious
	5	(5, 4]	(4, 3]	(3, 2]	(2, 1]
KPI dimension	Ecological level	Water abundance	Water quality	Water resource utilization	
	A1, A2, A3	B1, B2, B3, B4	C1, C2, C3	D1, D2, D3, D4, D5, D6	

Based on KPI assessment standard, the evaluation standard of index system is formulated by grade description method. The threshold of health grade is determined by consulting literature and experts and according to relevant national standards. According to the health status of each index, the specific index data is scored according to the grading threshold to get the health score of each index, and then the health score of each index is weighted and summed to get the overall evaluation result. The specific calculation equation is as follows:

10 $$H=\sum_{i=1}^{n}{h}_{i}{\omega }_{i}^{{\prime}},\quad i=\mathrm{1,2},...,n$$

In Eq. (10), H represents the total score of mine water circulation health evaluation. ${h}_{i}$ represents the health score of each index. ${\omega }_{i}$ represents the corresponding weight of each index.

In this article, an evaluation system for water circulation and utilization in mines under the IoT environment is established. The system encompasses the following aspects of utilization:

1. Supply–demand balance monitoring and evaluation system: This system uses the watershed or monitoring area as the basic evaluation unit and collects data to perform interpretive analysis. It particularly leverages remote sensing satellite data to accurately determine key factors related to water supply and demand analysis, such as the area and distribution of water sources in the monitoring area. Based on this, an analysis model is developed to assess the supply–demand balance in the monitoring and evaluation area. Key calculations and analyses include the annual total water supply, annual total water demand, and the balance difference between supply and demand. It also examines the supply volume and proportion of underground water, spring water, and reservoir water, along with the efficiency of various water sources and water engineering facilities.
2. Monitoring and evaluation system for water resource utilization efficiency in mines: The efficiency of water resource utilization in mines is divided into two categories: controllable channel water conveyance efficiency and agricultural water efficiency. The water use coefficient in the canal system can be calculated and analyzed using three methods: statistical analysis, monitoring of typical areas, and monitoring of fixed-point channel losses. The field water use coefficient is primarily obtained through monitoring the movement process of water in the field and analyzing water balance in typical areas. These two aspects are evaluated alongside monitoring and assessing water conversion supply and water consumption in mines.
3. Groundwater balance monitoring and evaluation system: Drawing on various monitoring and statistical data related to water resources, hydrology, meteorology, agriculture, etc., within the monitoring area, a groundwater balance model is established. This model analyzes groundwater recharge and discharge as well as groundwater storage variation within the monitoring area. Key calculations and analyses include the total groundwater recharge, total groundwater discharge, groundwater storage variation, and balance difference. The system also evaluates groundwater level changes within a year, interannual groundwater variations, groundwater extraction, spring overflow, groundwater depth at different depths and lithologies, and calculates groundwater evaporation.
4. Comprehensive evaluation system for the development and utilization of water resources in mines: Building upon the aforementioned sub-evaluation systems, an integrated evaluation system is developed for the comprehensive utilization of water resources in mine monitoring areas. This system serves to diagnose the rationality of watershed water circulation processes and the utilization of water resources in mines. The analysis and evaluation primarily focus on various perspectives, including water consumption for socioeconomic purposes and ecological environment, oasis water resource utilization efficiency, water supply guarantee rate, oasis water consumption structure and composition, ineffective and inefficient evaporation, water-salt balance status, urban water consumption and wastewater discharge, engineering support and utilization efficiency, water price and cost, water management, and groundwater level changes.

## Analysis

**Figure 12:**
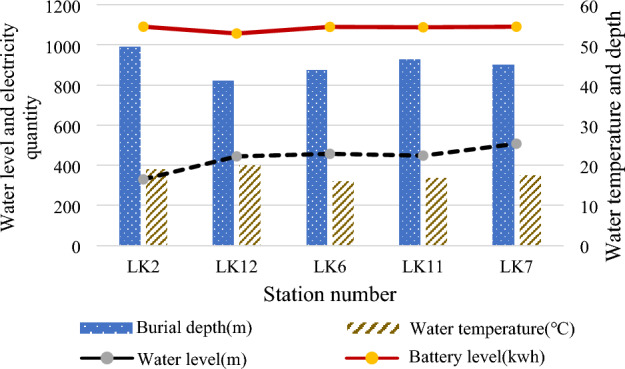
Ground substation data.

**Figure 13:**
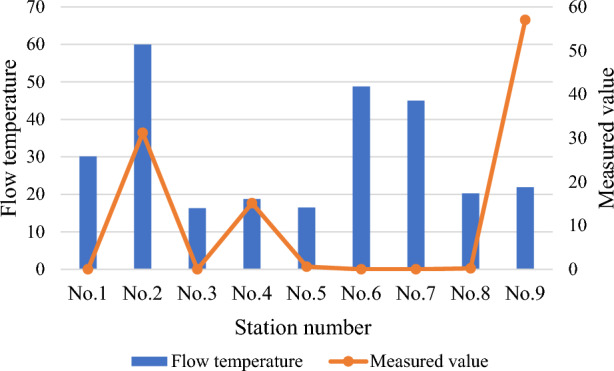
Data of underground substations.

First, the monitoring equipment is equipped at each monitoring point. Then, the ground data processing server and the client are installed and debugged. The server cabinet and the client computer are connected and powered by a network cable through the switch. The IP address is set. The monitoring software is configured on the client's computer. The system operation interface is shown in Figs. 12 and 13.

The micro-seismic and stress–strain monitoring systems are deployed at the working face. Meanwhile, the hydrological dynamic monitoring system is deployed at the main hydrological measurement points, such as hydrological long-view holes, rivers, underground drainage systems, and hydrological holes. After installation and commissioning, all systems run well and meet the monitoring requirements. The mine water monitoring and early warning big data platform has been successfully applied. It realizes multi-level networking, comprehensive scheduling, data integration, model analysis, and intelligent early warning. The actual application suggests that the mine water monitoring system works well. The system is comprehensive and achieves the expected goal. Therefore, it can be used as an essential reference for research and engineering of mine water monitoring and analysis technology.

**Figure 14:**
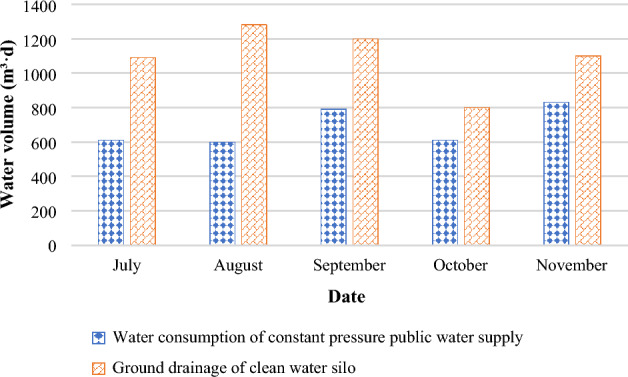
Water consumption of mine water discharge.

Figure 14 provides the water consumption data of fully mechanized mining, fully mechanized excavation working face, and road fire protection within the scope of the second division according to the 5-month water consumption statistics of the constant pressure water supply system (the data is quoted from the statistical ledger of the steady pressure water supply of a coal mining fleet).

The average daily water volume of constant pressure water supply is 715 m^3^; the average daily volume of sewage filtered in the goaf is 1685 m^3^. The cost of sewage treatment in the sewage treatment plant is 1.5 CNY/m^3^. It can save 3370 CNY per day for sewage treatment and 1072.5 CNY for lifting sewage, totaling 4442.5 CNY. The monthly cost savings is approximately 133,000 CNY, and the annual savings is approximately 1,599,000 CNY.

**Figure 15:**
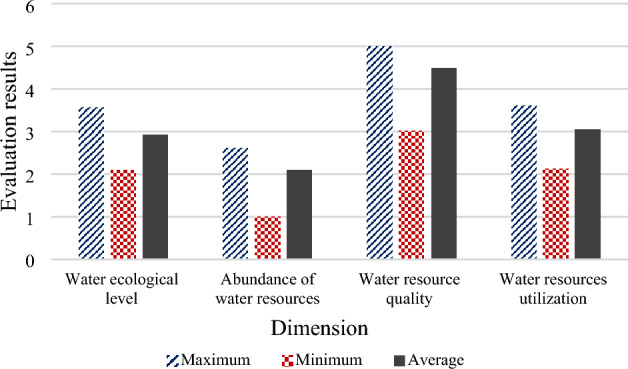
Scores of each dimension of KPI.

Standardization and dimensionless processing are carried out on each index data of water cycle utilization evaluation from 2016 to 2020 to reflect the evolution experience of the mine water cycle in China. The health evaluation analysis of the indicators is performed for each indicator and the given zoning criteria after treatment. Figure 15 presents the annual recycling situation of each indicator.

Water quality scored the highest in the dimensional evaluation, and the overall score was stable. The quality of water resources is healthy in all years, with a significant increase in 2020. This is mainly because all indicators in this dimension are free of serious pathologies. Besides, the water quality compliance rate of drinking water sources has been in a very healthy state. The water ecology level score shows an upward and better trend, with utilization moving from severe to more severe status in 2019–2020. Water use shifted from high to very low in 2016–2017, with the largest decrease. After that, water use rises. 2018–2019 all approached a lower state; 2020 reached a relatively normal state. Water abundance has the worst overall score. The score decreased in 2016–2017; it was in a poor condition in 2017 and 2018. Subsequent years show an upward trend, moving away from the inferior status.

**Figure 16:**
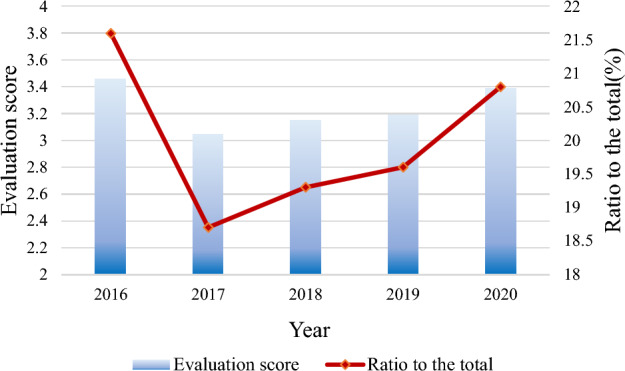
Evaluation of mine water recycling in 2016–2020.

Figure 16 represents the results of the year-by-year evaluation of water recycling for 2016–2020 based on the assessment of each indicator of the above survey.

It can be seen from Fig. 16 that the mine water recycling and utilization showed a relatively large downward trend from 2016 to 2020. According to the analysis of various indicators, the decline is mainly due to the low utilization rate of water resources development and the increase in domestic water consumption. Besides, the repetition rate of industrial water use is low. Correspondingly, some water resources are wasted, and the pressure on domestic and production water increases, putting the score in 2017 at the lowest point. 2017–2018 slowly grew with a 100% water quality compliance rate for drinking water sources. Each groundwater source is buried deep and is little susceptible to pollution. However, there has been a conflict between supply and demand. Water resources per capita and average water resources per acre are low; the water cycle evaluation score is 3.05–3.46 per year, which is always in good condition. In summary, improving the rationality of water resource development and utilization is the key to improving the water cycle’s various dimensions and overall balance.

Liu's research highlights the practical significance of studying the health evaluation of urban mine water circulation for urban planning, development, construction, and resource management. Using Xi'an City as a case study, Liu conducted an evaluation based on the concept of urban water circulation and the key attributes of water resources. From four dimensions, water ecological level, water resource abundance, water resource quality, and water resource utilization, 16 indicators were selected to construct a city water circulation health evaluation system based on key performance indicators^29^. However, the expected health status was not achieved with the establishment of this system. In this article, a comparison is made between the strengths and weaknesses of the indicator system established in that study. Specific evaluation indicators are derived based on water quality, water quantity, water use structure, water use efficiency, and hydrological and ecological environment, in alignment with the essence of water circulation. This indicator system exhibits a certain universality in the evaluation of water circulation and utilization in mines. Li evaluated the sustainable utilization of water resources in mines, categorizing the evaluation levels as “good, fairly good, average, poor”. The results revealed that the level of sustainable utilization of water resources in mines was "average". In some areas, the water resources in mines were in a pathological state and had not been alleviated^30^. Building upon the premise that water resources in mines still exhibited a sub-healthy state, this article evaluated the mine water circulation and utilization from 2016 to 2020. The findings indicated a gradual decline in the mine water circulation and utilization over the years. The primary factors contributing to this decline were the lower utilization rate of water resources, an increased rate of water consumption for residential purposes, and a relatively low repetition rate of industrial water use, resulting in some wastage of water resources. It is evident that improving the rationality of water resource development and utilization in mines is crucial for enhancing various dimensions and the overall balance of water circulation.

## Conclusion

The implementation and operation of the smart mine water resource network mark a solid step in building intelligent mines. This network fully combines network technology and on-site equipment, maximizes the rational allocation of resources, reduces the workers’ labor intensity, and improves the working environment of workers. In addition, it transforms the positions of front-line operators into inspections and processing and appropriately reduces front-line staffing, achieving reduced staff and increased efficiency. It has become possible to use technology to build cooling, dust removal, and external discharge systems and dynamically monitor water utilization and evaluation via IoT. Applying the latest technology will profoundly affect the water control work in the mines threatened by old empty water, surface water, roof, and floor water hazards. Common technologies include automatic collection and transmission of measuring point data, intelligent report generation, graphic visualization of hydrological data, and wise early warning and cloud platform. They also have demonstration and reference significance and promotion role for China's coal mine water recycling.

Still, there are some shortcomings. The construction of the indicator system takes a relatively short period of time. Therefore, the workload and research depth of the research are relatively limited. It cannot reflect a comprehensive effect. Future research will verify the scientific validity of the index system and improve relevant ideas and methods. Moreover, breakthroughs in aperiodic time series are insufficient for using existing mine water inflow prediction algorithms. Currently, the relevant theory and technology of neural networks are developing rapidly. Future work can combine the theory and method related to neural networks in the direction of data processing and analysis to meet high requirements.

## Data Availability

All data generated or analysed during this study are included in this published article[and its supplementary information files]. If someone wants to request the data from this study please contact the Corresponding author.(Yang Li, e-mail: dly_1@163.com).
